# Supplementary material for: One Health collaboration is more effective than single-sector actions at mitigating SARS-CoV-2 in deer
Source: Nat Commun. 2024 Oct 7;15:8677. doi: 10.1038/s41467-024-52737-0 (PMC11458903; doi:10.1038/s41467-024-52737-0)
Supplement: Supplementary file 1 — Supplementary Information [file 41467_2024_52737_MOESM1_ESM.pdf]

Supplementary Information

Title: One Health collaboration is more effective than single sector actions at mitigating SARS-CoV-2 in deer

Author list:
JONATHAN D. COOK[1,†], ELIAS ROSENBLATT[2], GRAZIELLA V. DiRENZO[3], EVAN H. CAMPBELL GRANT[4], BRITTANY A. MOSHER[2], FERNANDO ARCE[5], SONJA A. CHRISTENSEN[6], RIA R. GHAI[7], and MICHAEL C. RUNGE[1]

Affiliations:
[1] *U. S. Geological Survey, Eastern Ecological Science Center, Laurel, MD, USA*
[2] *University of Vermont, Rubenstein School of Environment and Natural Resources, Burlington, VT, USA*
[3] *U. S. Geological Survey, Massachusetts Cooperative Fish and Wildlife Research Unit, University of Massachusetts, Amherst, MA, USA*
[4] *U. S. Geological Survey, Eastern Ecological Science Center, Turner's Falls, MA, USA*
[5] *University of Massachusetts, Amherst, Department of Environmental Conservation, MA, USA*
[6] *Michigan State University, Department of Fisheries and Wildlife, East Lansing, MI, USA*
[7] *U.S. Centers for Disease Control and Prevention, Atlanta, GA, USA*

[†]Corresponding author: Jonathan D. Cook (Contact: jcook@usgs.gov)

Supplementary Table 1. Guidance Committee Member Names, sector, and affiliations of members of the guidance committee for this study

**Committee Member**	**Sector**	**Agency**
Darlene Konkle	Agricultural	Wisconsin Department of Agriculture, Trade, and Consumer Protection
Roxanne Mullaney	Agricultural	U.S. Department of Agriculture
Susan Rollo	Agricultural	Texas Department of State Health Services
Jennifer Siembieda	Agricultural	U.S. Department of Agriculture
Nora Wineland	Agricultural	Michigan Department of Agriculture and Rural Development
Samantha Gibbs	Wildlife	U.S. Fish and Wildlife Service
Colin Gillin	Wildlife	Oregon Department of Fish and Wildlife
Allen Gosser	Wildlife	U.S. Department of Agriculture
Darby Murphy	Wildlife	U.S. Fish and Wildlife Service
Paul Johansen	Wildlife	West Virginia Department of Natural Resources
Lisa Shender	Wildlife	National Park Service
Jason Sumners	Wildlife	Missouri Department of Conservation
Michael Tonkovich	Wildlife	Ohio Department of Natural Resources
Casey Barton Behravesh	Public Health	Centers for Disease Control and Prevention
Colin Basler	Public Health	Centers for Disease Control and Prevention
Ria Ghai	Public Health	Centers for Disease Control and Prevention
Chelsea Gridley-Smith	Public Health	National Association of County and City Health Officials

Supplementary Notes

Alternatives

Complete list of alternative actions that may be effective at mitigating SARS-CoV-2 introduction and spread in captive or wild white-tailed deer populations.

1. Manipulate wild populations or habitat conditions.
    a. Local reductions in wild deer populations through agency culling or hunter harvest.
    b. Prohibit deer feeding and baiting to reduce congregations of wild deer.
2. Restrictions on research, survey, monitoring or management.
    a. Pause/reduce research permits for work on wild deer or other susceptible wildlife/animal species.
3. Education and outreach. Intended to communicate the risk that SARS-CoV-2 presents to humans, wildlife, domestic and companion animals. Can be coordinated through veterinarians, public health officials, regulators, hunter's digest materials, local neighborhood web-apps. Can also be targeted based on human disease trends, wildlife surveillance activities.
    a. Encourage proper deer processing activities (field dressing).
    b. Encourage limiting human-deer interactions including participating in conduct that attracts wildlife.
    c. Encourage pet owners to keep animals indoors.
    d. Encourage (and train on the proper) use of enhanced personal protective equipment during interactions with captive and wild deer
4. Alter/Enhance captive, farming, exhibition, or rehabilitation practices.
    a. Depopulation
    b. Implement new accreditation program or otherwise enhanced testing
    c. Animal vaccination requirement
    d. Isolation requirements within facilities or from external environments
        i. Double fencing
        ii. Enhanced inspections of barriers
    e. Limiting interactions between humans and deer (expand focus beyond human-animal welfare to include disease-related enhancements)
    f. Limit release of animals
    g. Animal food storage and distribution requirements to reduce animal congregations and mixing (biosecurity)
    h. Require enhanced ventilation in enclosed agricultural/zoo/captive settings
    i. Enhanced PPE or vaccination for humans
    j. Potential active measures: test and cull, isolate and test, or other restrictions on trade/translocation.
5. Reduce human-wildlife interactions
    a. Ban feeding and baiting of wildlife, specifically wild deer, as part of hunting or other wildlife viewing activities.
    b. Temporary closures of public trails, parks, other locations.
    c. Hazing activities near high-risk areas of human-deer contact, such as wastewater treatment ponds.

d. Regulations on carcass disposal for processors, taxidermists, and other agricultural or hunting activities.

Overview of guidance committee process

To generate the decision framing elements (problem scoping, fundamental objectives, causal chain, and alternatives) that were necessary to evaluate SARS-CoV-2 in white-tailed deer, we convened a 17-member guidance committee (Supplementary Table 1) and held a total of 10 facilitated meetings. The focus of the first seven meetings is described below. The remaining three meetings were focused on presentation of results.

Meeting 1: Introductions, project overview, and desirable outcomes (Committee focus)

Focus:

1) Introductions
2) Project Overview
    a. Project goals
    b. Timeline
    c. Committee composition
        i. Membership and Roles
3) Desirable outcomes of management decisions
    a. What are the outcomes that people care most about?

Outcomes:

1) Understanding of project
2) Understanding desirable outcomes of management decisions surrounding SARS-CoV-2 risks to humans, white-tailed deer, or closely associated species.

Meeting 2: Hypotheses of transmission (Committee focus)

Focus:

1) Developing a shared understanding of the scope of the problem
    a. Brainstorm hypotheses on how human-deer transmission could occur

Outcomes:

1) Draft list of hypotheses of how human-deer transmission might occur

Meeting 3: Hypotheses of transmission (Sector focus)

Focus:

1) Use draft list of hypotheses to develop a conceptual understanding of system

Outcomes:

1) Draft conceptual diagram of sector-specific disease system

Meeting 4: Hypotheses of transmission cont. (Sector focus)

Focus:

1) Refine conceptual diagram of sector-specific disease system

Outcomes:

1) Finalized conceptual diagram of sector-specific disease system

Meeting 5: Hypotheses of transmission cont. (Committee focus)
Focus:
1) Present complete conceptual diagram of disease system in Committee
2) Brainstorm points of intervention

Outcomes:
1) Suggested changes to conceptual diagram
2) Draft list of interventions

Meeting 6: Systems map with points of intervention (Committee focus)
Focus:
1) Finalize conceptual diagram of disease system
2) Present potential points intervention
3) Open discussion on interventions

Outcomes:
1) Complete conceptual map and points of intervention

Meeting 7: Unresolved tasks/Review/Coordination (Committee focus)
Focus:
1) Address any remaining issues that arose during mapping meetings
2) Make plan for dissemination of findings
3) Develop recommendations for future needs

Outcomes:
1) A plan for distribution of findings
2) List of future needs
