# Supplementary material for: One Health collaboration is more effective than single-sector actions at mitigating SARS-CoV-2 in deer
Source: Nat Commun. 2024 Oct 7;15:8677. doi: 10.1038/s41467-024-52737-0 (PMC11458903; doi:10.1038/s41467-024-52737-0)
Supplement: Supplementary file 2 — Reporting Summary [file 41467_2024_52737_MOESM2_ESM.pdf]

nature portfolio

Corresponding author(s): Jonathan D. Cook

Last updated by author(s): Sep 12, 2024

Reporting Summary

Nature Portfolio wishes to improve the reproducibility of the work that we publish. This form provides structure for consistency and transparency in reporting. For further information on Nature Portfolio policies, see our Editorial Policies and the Editorial Policy Checklist.

Statistics

For all statistical analyses, confirm that the following items are present in the figure legend, table legend, main text, or Methods section.

n/a	Confirmed	
☐	☒	The exact sample size (*n*) for each experimental group/condition, given as a discrete number and unit of measurement
☒	☐	A statement on whether measurements were taken from distinct samples or whether the same sample was measured repeatedly
☒	☐	The statistical test(s) used AND whether they are one- or two-sided<br>*Only common tests should be described solely by name; describe more complex techniques in the Methods section.*
☒	☐	A description of all covariates tested
☒	☐	A description of any assumptions or corrections, such as tests of normality and adjustment for multiple comparisons
☐	☒	A full description of the statistical parameters including central tendency (e.g. means) or other basic estimates (e.g. regression coefficient) AND variation (e.g. standard deviation) or associated estimates of uncertainty (e.g. confidence intervals)
☒	☐	For null hypothesis testing, the test statistic (e.g. *F*, *t*, *r*) with confidence intervals, effect sizes, degrees of freedom and *P* value noted<br>*Give P values as exact values whenever suitable.*
☒	☐	For Bayesian analysis, information on the choice of priors and Markov chain Monte Carlo settings
☒	☐	For hierarchical and complex designs, identification of the appropriate level for tests and full reporting of outcomes
☒	☐	Estimates of effect sizes (e.g. Cohen's *d*, Pearson's *r*), indicating how they were calculated

Our web collection on statistics for biologists contains articles on many of the points above.

Software and code

Policy information about availability of computer code

Data collection	All code and generated data used in this study are available in the R software package whitetailedSIRS. Citation: Rosenblatt, E, Rudolph, J.F., Arce, F., Cook, J. D., DiRenzo, G.V., Grant, E.H.C., Runge, M.C., and Mosher, B.A., 2023. whitetailedSIRS: A package to project SARS-CoV-2 outbreak dynamics in white-tailed deer. Version 1.0.0: U.S. Geological Survey software release, https://doi.org/10.5066/P9TZK938
Data analysis	All code and generated data used in this study are available in the R software package whitetailedSIRS. Citation: Rosenblatt, E, Rudolph, J.F., Arce, F., Cook, J. D., DiRenzo, G.V., Grant, E.H.C., Runge, M.C., and Mosher, B.A., 2023. whitetailedSIRS: A package to project SARS-CoV-2 outbreak dynamics in white-tailed deer. Version 1.0.0: U.S. Geological Survey software release, https://doi.org/10.5066/P9TZK938

For manuscripts utilizing custom algorithms or software that are central to the research but not yet described in published literature, software must be made available to editors and reviewers. We strongly encourage code deposition in a community repository (e.g. GitHub). See the Nature Portfolio guidelines for submitting code & software for further information.

Data

Policy information about availability of data

All manuscripts must include a data availability statement. This statement should provide the following information, where applicable:

- Accession codes, unique identifiers, or web links for publicly available datasets
- A description of any restrictions on data availability
- For clinical datasets or third party data, please ensure that the statement adheres to our policy

All code and generated data used in this study are available in the R software package whitetailedSIRS. Citation: Rosenblatt, E, Rudolph, J.F., Arce, F., Cook, J. D., DiRenzo, G.V., Grant, E.H.C., Runge, M.C., and Mosher, B.A., 2023. whitetailedSIRS: A package to project SARS-CoV-2 outbreak dynamics in white-tailed deer. Version 1.0.0: U.S. Geological Survey software release, https://doi.org/10.5066/P9TZK938

Research involving human participants, their data, or biological material

Policy information about studies with human participants or human data. See also policy information about sex, gender (identity/presentation), and sexual orientation and race, ethnicity and racism.

Reporting on sex and gender	None of this research involved human participants, their data, or biological material.
Reporting on race, ethnicity, or other socially relevant groupings	None of this research involved human participants, their data, or biological material.
Population characteristics	None of this research involved human participants, their data, or biological material.
Recruitment	None of this research involved human participants, their data, or biological material.
Ethics oversight	None of this research involved human participants, their data, or biological material.

Note that full information on the approval of the study protocol must also be provided in the manuscript.

Field-specific reporting

Please select the one below that is the best fit for your research. If you are not sure, read the appropriate sections before making your selection.

☐ Life sciences ☐ Behavioural & social sciences ☒ Ecological, evolutionary & environmental sciences

For a reference copy of the document with all sections, see nature.com/documents/nr-reporting-summary-flat.pdf

Ecological, evolutionary & environmental sciences study design

All studies must disclose on these points even when the disclosure is negative.

Study description	This was a multidisciplinary study designed to better understand and mitigate SARS-CoV-2 spread in white-tailed deer across One Health sectors. We worked with a One Health committee to identify risks associated with SARS2 in deer, the objectives of agencies that were affected by the spread of disease in white-tailed deer, and the alternative actions they might take to mitigate those risks. We then developed a dynamic compartmental model that linked wild and captive deer herds and humans and simulated SARS-CoV-2 dynamics. We then tested single-sector alternatives alone and in combination with other sector actions. Our results are reported as means and prediction intervals for each treatment with no tests of statistical significance.
Research sample	No research samples were taken, however, the study was designed to simulate wild and captive white-tailed deer dynamics (including behavior and movement dynamics) to evaluate the potential spread of a zoonotic disease. We determined sample size (number of simulations) based on preliminary evaluation of the data, the ability to fully explore parameter space including interactions, and used the sensitivity to model results for a range of samples sizes to guide this decisions.
Sampling strategy	No sampling strategy was employed in this study.
Data collection	Data was collected using a literature review and formal expert elicitation protocols. The expert elicitation protocols were described in detail in Rosenblatt et al. which is a companion study to this paper.
Timing and spatial scale	No data were collected; however, the data simulations were projected across 120 days in each scenario to incorporate fall deer behavior (September-December). We focused on the fall season as deer reproductive behavior results in increased deer contact rates and multiple hunting seasons and seasonal captive activities could increase human-to-deer interactions.
Data exclusions	No data were excluded.
Reproducibility	All code and generated data used in this study are available in the R software package whitetailedSIRS. Citation: Rosenblatt, E,

Reproducibility	Rudolph, J.F., Arce, F., Cook, J. D., DiRenzo, G.V., Grant, E.H.C., Runge, M.C., and Mosher, B.A., 2023. whitetailedSIRS: A package to project SARS-CoV-2 outbreak dynamics in white-tailed deer. Version 1.0.0: U.S. Geological Survey software release, https://doi.org/10.5066/P9TZK938 . Included in the release are vignettes that allow researchers to reproduce our analyses and results.
Randomization	n/a
Blinding	n/a

Did the study involve field work? ☐ Yes ☒ No

Reporting for specific materials, systems and methods

We require information from authors about some types of materials, experimental systems and methods used in many studies. Here, indicate whether each material, system or method listed is relevant to your study. If you are not sure if a list item applies to your research, read the appropriate section before selecting a response.

Materials & experimental systems

n/a	Involved in the study	
☒	☐	Antibodies
☒	☐	Eukaryotic cell lines
☒	☐	Palaeontology and archaeology
☒	☐	Animals and other organisms
☒	☐	Clinical data
☒	☐	Dual use research of concern
☒	☐	Plants

Methods

n/a	Involved in the study	
☒	☐	ChIP-seq
☒	☐	Flow cytometry
☒	☐	MRI-based neuroimaging

Plants

Seed stocks	*Report on the source of all seed stocks or other plant material used. If applicable, state the seed stock centre and catalogue number. If plant specimens were collected from the field, describe the collection location, date and sampling procedures.*
Novel plant genotypes	*Describe the methods by which all novel plant genotypes were produced. This includes those generated by transgenic approaches, gene editing, chemical/radiation-based mutagenesis and hybridization. For transgenic lines, describe the transformation method, the number of independent lines analyzed and the generation upon which experiments were performed. For gene-edited lines, describe the editor used, the endogenous sequence targeted for editing, the targeting guide RNA sequence (if applicable) and how the editor was applied.*
Authentication	*Describe any authentication procedures for each seed stock used or novel genotype generated. Describe any experiments used to assess the effect of a mutation and, where applicable, how potential secondary effects (e.g. second site T-DNA insertions, mosiacism, off-target gene editing) were examined.*
